# The Evaluation of the Inner Diameter of the Airway in Asthma Recovery by using HRCT: A Retrospective Observational Cohort Study

**DOI:** 10.2174/0115734056424104251026155433

**Published:** 2025-11-28

**Authors:** Wenping Fan, Yu Luo, Zhiye Chen

**Affiliations:** 1 Department of Radiology, Hainan Hospital of PLA General Hospital, Sanya, Hainan 572013, China

**Keywords:** HRCT, Airway inner diameter, Asthma, Segmental bronchi, Lobar bronchi, PFT

## Abstract

**Background:**

Although airway size changes occur in patients with chronic asthma, HRCT has not yet been used to assess changes in the inner diameter of the airways.

**Objective:**

This study aimed to evaluate the airway diameter in asthma recovery by using HRCT.

**Methods:**

Thirty patients with asthma were recruited and underwent HRCT examination in acute exacerbation and stable phase, respectively. The inner diameter of the airway (Din) was measured from the bilateral main bronchi to all 18 segmental bronchi during acute exacerbation and the stable phase.

**Results:**

The inner diameter of the airway reduced significantly in the acute exacerbation period compared to the stable period (*P* < 0.05). The mean Din reduction (%) in segmental bronchi was 12% and that in lobar bronchi was 6%. Among the 30 patients, the dorsal segmental bronchi of both lower lobes showed the highest incidence of stenosis during acute exacerbation compared to the stable phase (right: 18 cases; left: 16 cases), while the lingular bronchus exhibited the highest stenosis incidence among lobar bronchi (18 cases). Although the number of stenotic segmental and lobar bronchi demonstrated a positive correlation with disease severity across mild, moderate, and severe groups, no statistically significant differences were observed in intergroup comparisons (*P*>0.05).

**Discussion:**

HRCT showed generalized airway narrowing during asthma exacerbation, which reversed with treatment. The lack of correlation with FEV1% may have resulted from the heterogeneity of airway remodeling, the static nature of CT assessment, and the disproportionate functional impact of key airway stenosis. This underscores the complementary value of anatomical imaging alongside functional testing.

**Conclusion:**

CT images of bronchial stenosis showed obvious dilation after appropriate medication, and the inner diameter of the airway can be used as a practical and convenient index to evaluate the recovery of asthma.

## 1 INTRODUCTION

Asthma is a global health issue affecting all age groups, with its prevalence continuing to rise and imposing a significant social burden. Epidemiological data show that asthma affects 262 million people worldwide, with 15-20% of the population in many countries suffering from the disease, which significantly impacts labor productivity and family life [1-3].

Asthma presents with clinical symptoms, such as shortness of breath, cough, and wheezing during exacerbations, and is characterized by reversible airflow limitation, airway hyperresponsiveness, chronic inflammation, and airway remodeling [4, 5]. These pathological changes not only affect the peripheral small airways, but also affect the entire lower respiratory tract [6]. High-resolution CT (HRCT), with its superior resolving power, can detect imaging features as small as 100-200 μm and assess small airway regions with diameters of 1.5-2.0 mm, making it a valuable tool for evaluating disease severity and structural changes in asthma [7].

Recently, some studies have reported changes in airway dimensions in patients with chronic asthma during acute exacerbation, such as luminal area and airway wall area, used to assess airway narrowing and remodeling [8, 9]. However, few studies have focused on the inner diameter of the airway, except for a study that reported a decreased airway diameter to be notably observed in segmental bronchi and their next several generations during asthma exacerbation compared to the stable phase [10].

In this study, we evaluated changes in the inner diameter of the airway (Din) from lobar bronchi to segmental bronchi on HRCT images between the acute exacerbation and stable phases, with the hope of providing additional approaches to asthma assessment.

## 2 MATERIALS AND METHODS

### 2.1 Study Population

This retrospective study was conducted in accordance with the ethical principles outlined in the Declaration of Helsinki and was approved by the Institutional Review Board of Hainan Hospital, PLA General Hospital. This study included 30 patients (female/male = 13/17, age: 38.7 ± 11.9 years) with asthma who were admitted to our hospital from June 2022 to September 2024. The patients were divided into a mild severity group (n = 12), a moderate severity group (n = 9), and a severe severity group (n = 9), according to the severity of the acute attack. The details of the patients can be found in Table **1**. The inclusion criteria for all patients were as follows: (1) a history of typical symptoms; (2) an improvement rate of FEV1 > 12% between the acute phase and the stable phase; (3) no concurrent other lung diseases, such as emphysema and bronchiectasis, and no severe asthma cases. The exclusion criteria were as follows: (1) age < 18 or > 80 years; (2) suffering from severe diseases of vital organs, such as the heart, liver, and kidneys; (3) cancer patients; (4) patients with mental illness or mental cognitive impairment.

All patients were tested 20 minutes before and 20 minutes after inhaling 200 μg of salbutamol by nebulization during the acute phase and the stable phase. The post-bronchodilation values in the stable phase were used as the baseline.

### 2.2 CT Data Acquisition and Reconstruction

All patients were scanned with a 128-slice CT scanner (SOMATOM, Definition, Erlangen, Germany). The CT scans were performed using the following parameters: tube voltage: 120 kV; tube current: automatic mA modulation (SmartmA); pitch: 1.375 mm; slice thickness: 1.25 mm; slice interval: 1.0 mm; field of view (FOV): 36 cm. CT scans were obtained at full inspiration in the supine position; all patients were carefully instructed on breath-hold maneuvers and could hold their breath for a moment during asthma exacerbation. Reconstructed data were transferred to the workstation and analyzed with an IntelliSpacePortal V10.1 software program (Royal Philips, Netherlands). The Din was measured from the bilateral main bronchi to all 18 segmental bronchi. As shown in Fig. (**1**), we first selected the target bronchus and measured the Din value using the software, and we obtained the mean Din value of the target bronchus. The same procedure was performed during the exacerbation and stable phases, and abnormal values were excluded if the airway was obstructed by a mucus plug or was absent congenitally.

### 2.3 Pulmonary Function Tests

Pulmonary function tests (PFTs) and CT scans were performed at the same period, both in the exacerbation and stable phases. Using a routine pulmonary function testing instrument (MasterScreen Pneumo; Jaeger, Germany), we measured forced expiratory volume in one second (FEV1) and forced vital capacity (FVC). FEV1 (% predicted) was obtained according to the prediction equations of the Japanese Respiratory Society [9].

### 2.4 Statistical Analysis

The mean Din of all lobar and segmental bronchi was calculated in all 30 patients, and the data have been presented as mean±SD. All statistical analysis was performed using the GraphPad Prism 7 software (GraphPad Software, Inc., La Jolla, CA, USA). The values were compared between the acute exacerbation and the stable phase. Comparison within groups was performed with paired t-tests, and comparison across multiple groups was performed with one-way ANOVA. The correlation between the incidence of narrowing bronchi and FEV1 (% predicted) was analyzed by using the Pearson method. A *P*-value set as <0.05 was considered statistical difference.

## 3 RESULT

### 3.1 Comparison of Acute Exacerbation and Stable Phase Bronchial Stenosis Rates

In contrast to the steady phase, patients' airway diameters were noticeably smaller during the acute exacerbation. The dorsal segmental bronchi of the lower lobes showed the highest incidence of stenosis (right: 18 cases; left: 16 cases), with a mean diameter reduction of 12% for segmental bronchi (Table **2**). The lingular bronchus showed the highest prevalence of stenosis (18 cases), and the mean reduction in diameter for lobar bronchi was 6% Table (**3**). However, there was no discernible relationship between the projected FEV1% and the incidence of stenosis (R^2^ = 0.001, *P* > 0.05).

### 3.2 Comparison of Bronchial Stenosis Numbers among Mild, Moderate, and Severe Groups

The number of stenotic bronchi varied across disease severity groups. In the mild group, the average number of stenotic segmental bronchi was 4.9±3.1, while the moderate and severe groups had 5.0±3.7 and 6.2±3.0, respectively. For lobar bronchi, the average number of stenotic bronchi was 3.0±2.6 in the mild group, 3.9±3.1 in the moderate group, and 4.7±2.9 in the severe group, respectively (Fig. **2**). Although a positive correlation was observed between the number of stenotic bronchi and disease severity, intergroup comparisons showed no statistically significant differences (*P*>0.05).

## 4 DISCUSSION

In this study, changes in the airway inner diameter of asthma patients were evaluated using an automated quantitative software system, and, for the first time, the phenomenon of generalised narrowing of the airways from the main bronchi to the segmental bronchi during the acute phase was revealed. The main findings included the following: (1) the inner diameters of the airways at all levels were significantly narrowed during the acute attack phase compared to those in the stable phase; (2) the dorsal segmental bronchi of the middle and lower lobes and the bronchus of the lingual lobe had the highest rates of stenosis within their respective groups; (3) there was no significant correlation and statistical significance between the number of narrowed bronchi and the predicted value of FEV1%.

The inner diameters of airways at all levels during the acute attack phase were significantly constricted compared to those during the stable phase, which may be related to the pathophysiological mechanism of asthma. Asthma is a chronic inflammatory disease of the airways, in which many cells and cellular components play important roles in the inflammatory process, especially mast cells, eosinophils, T lymphocytes, neutrophils, and epithelial cells [11]. Under the action of external triggers, the cytokines produced by the mediation of inflammatory cells during an asthma attack may cause mucus production, tissue edema, and smooth muscle contraction, and these three mechanisms lead to the clinical symptoms of airway stenosis in asthma patients during an acute attack [12].

The high stenosis rates of the dorsal segmental bronchi of the lower lobe and the bronchus of the lingular lobe in this study may be related to their special anatomical positions. The former has a steep running angle, and the latter has a relatively narrow lumen, making them more susceptible to the influence of inflammatory edema. However, it is worth noting that there is significant heterogeneity among individuals; some patients can develop severe symptoms even with only a few bronchi affected, suggesting that the stenosis of local key airways may have a magnifying effect on the overall pulmonary function.

In this study, no significant correlation or statistical association was observed between the number of narrowed bronchi and FEV1% predicted values. Firstly, this discrepancy may be attributed to the heterogeneity of airway remodeling in asthma, characterized by structural alterations, including epithelial fibrosis, increased smooth muscle mass, submucosal gland hypertrophy, vascular proliferation, and epithelial changes, which typically develop during prolonged chronic injury in asthma [13]. Although several cell types play a role in asthma, it is assumed that the smooth muscles of the airways and epithelial cells play an important role. Changes in airway smooth muscle mass are particularly evident in the large airways of asthma patients [14]. James *et al*. also supported this view in their model of airway narrowing and found that the heterogeneity of central airway narrowing can lead to abnormal ventilation in the structurally normal distal lung [15]. The narrowing of some major airways may lead to disproportionate functional impairment. Therefore, simply counting the number of narrowed airways may not accurately reflect their functional impact on overall airway resistance. Otherwise, the delta lumen phenomenon, defined by variations in luminal area from the third- to sixth-generation equivalent airway segments, was first described in asthmatic airway remodeling by Shim *et al*. [16]. This phenomenon demonstrates that bronchial lumina may appear normal on CT imaging during full inspiration but exhibit significant narrowing during complete expiration [17]. Consequently, the fundamental differences between static CT-based assessments and dynamic pulmonary function testing may account for the observed results in this study. Furthermore, previous studies have identified distinct patterns of airway-pulmonary function correlations depending on disease phenotype. In stable asthma patients, the luminal diameter of fifth-generation bronchi demonstrates the strongest association with pulmonary function parameters, whereas in asthma patients with emphysema, sixth-generation bronchial diameters correlate more significantly with lung function [18]. Finally, in the present study, our measurements focused on second- and third-generation bronchi, which may represent another contributing factor to the observed lack of correlation between airway dimensions and FEV1% predicted values.

Pulmonary function tests have been the standard test for the diagnosis of asthma [19]. However, they require close patient cooperation and take a long time to conduct. Moreover, the test has the potential to rupture pulmonary bullae, worsening the underlying disease. Thus, we need more methods to evaluate lung diseases, such as asthma. With advances in high-resolution computed tomography (HRCT), it has become a valuable tool for diagnosing lung diseases. We were able to obtain the objective parameters of the airway with the quantitative analysis of imaging. Previous studies have reported that the ratio of the airway wall area (WA) to body surface area (BSA) correlates with pulmonary function measurement, and the correlation coefficients between CT parameters and airflow limitation increase when the airway size decreases [4]. Airway wall thickness had a positive effect on the degree of airflow limitation in asthma [20]. Yuki and colleagues demonstrated that segmental and subsegmental bronchi were significantly obstructed during acute exacerbation, as evidenced by decreased airway diameter and mucus plugs, which had been recently recognized as another CT parameter for the severity of asthma and could be clearly shown on CT images [10]. In this study, we further demonstrated a decreased Din of the larger bronchi during acute exacerbation, which could be directly seen on the CT image or measured by quantitative analysis. It supported the previous observation that the entire lower airway is involved in asthma inflammation. Our data also showed that for individual patients, not every bronchus was significantly narrowed during acute asthma exacerbation, and that a small part of the narrowed bronchi could lead to a severe manifestation.

## 5 LIMITATION

There were some limitations in the study that need to be considered. First, the Din can be influenced by many factors. For example, infection, pleural effusion, etc., were excluded in our study, but these are common complications in clinical practice and might have effects on the diameter of the airway. Next, life-threatening patients were ruled out in this study because they could not undergo a CT scan. Thus, the parameters of the bronchi in these patients were not available. Moreover, dynamic evaluation of Din was needed in asthma patients; otherwise, it would have been hard to recognize airway stenosis on a single CT scan.

## CONCLUSION

Based on HRCT assessment, this study showed that acute asthma exacerbations cause significant, reversible narrowing of lobar and segmental bronchi, especially in the dorsal lower lobe and the lingular segments. The findings demonstrated widespread airway involvement in asthma, including the central airways. HRCT-based inner diameter measurement provided a practical tool for evaluating structural airway changes and complemented pulmonary function tests. Future studies should establish links between specific narrowing patterns and clinical outcomes to guide personalized treatment.

## Figures and Tables

**Fig. (1):**
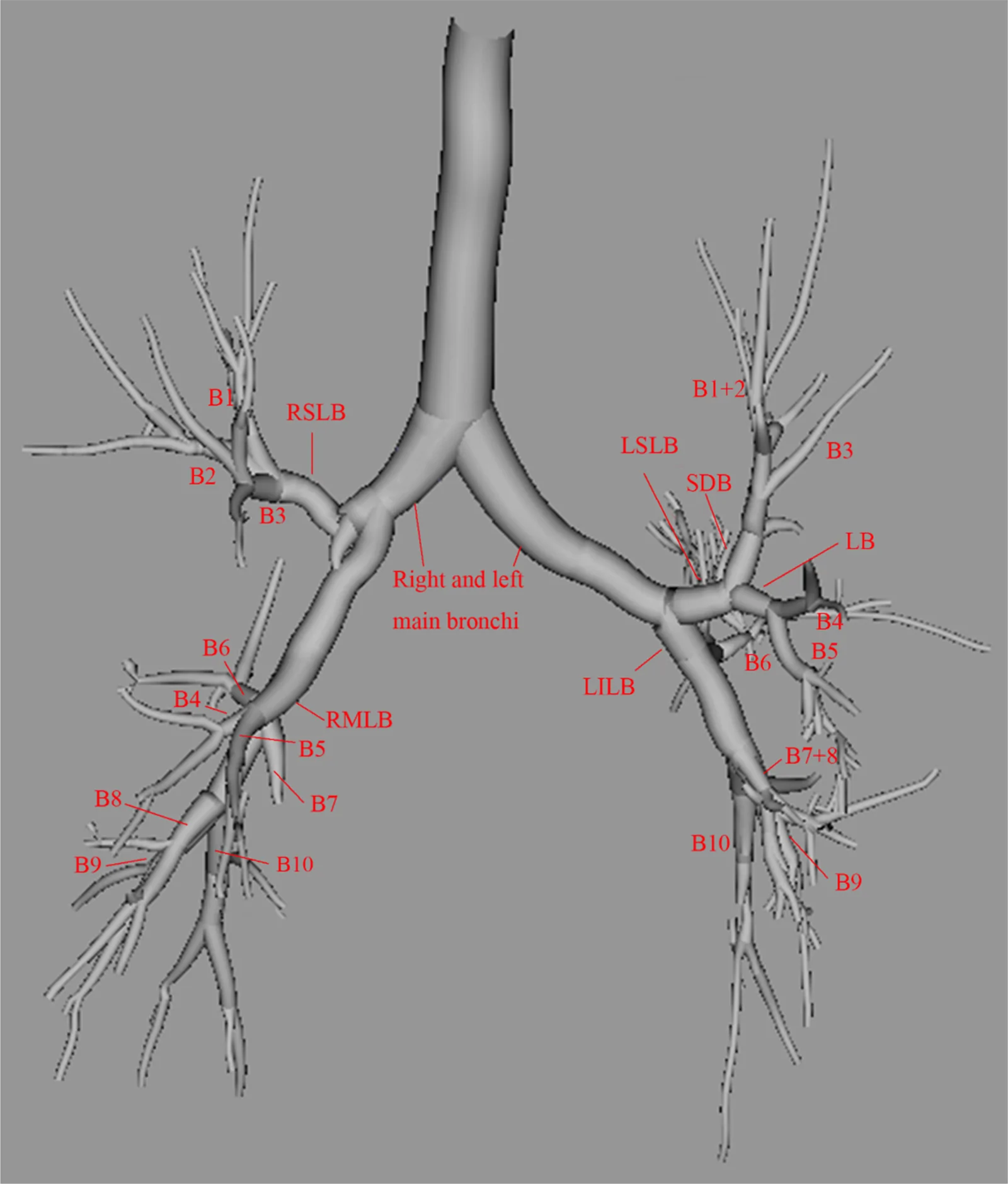
The representative picture and abbreviation of the bronchial tree used in this study. **Abbreviations:** RSLB, Right superior lobar bronchi; RMLB, Right middle lobar bronchi; SDB, Superior division bronchi; LSLB, Left superior lobar bronchi; LILB, Left inferior lobar bronchi; B, Bronchi.

**Fig. (2):**
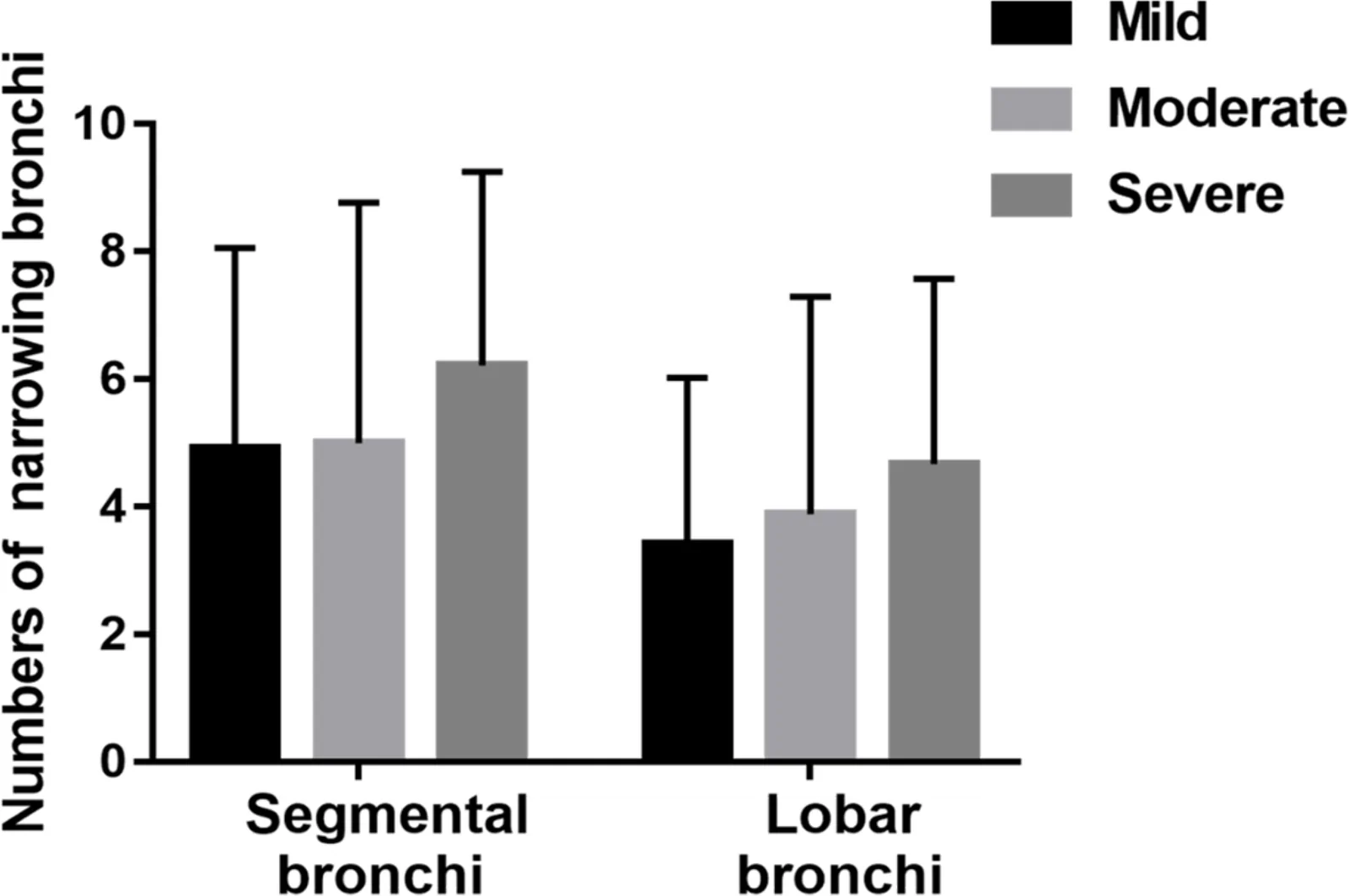
The number of stenotic segmental and lobar bronchi varied with disease severity; the severe group had the highest averages (6.2±3.0 for segmental, 4.7±2.9 for lobar).

**Table 1:** Characteristics of patients.

Number of Subjects	30
Age (years)	38.7±11.9
Gender (F:M)	13:17
Disease duration (years)	5.7±4.5
Smoking history (number)	9
Body mass index (Kg/m^2^)	23.5±4.5
FEV1% (%predicted)	76.0±18.0
***Exacerbation severity (number)***	
	Mild: 12
	Moderate: 9
	Severe: 9
***Asthma medication***	
	TP: 5
	LABA: 13
	LTRA: 9
	ICS: 28
	OCS: 9

Data are presented as mean±SD. FEV1: Forced expiratory volume in 1 second.

**Table 2:** The changes of chest CT-estimated segmental bronchial luminal Din between acute exacerbation and stable phase of asthma.

Segmental Bronchi	Exacerbation	Stable Phase	Reduction of Din (%)	Incidence of Stenosis
LB1+2	3.64 (0.86)	4.39 (0.87)	15%***	15
LB3	4.11 (1.07)	4.70 (0.77)	12%***	13
LB4	3.38 (0.79)	3.88 (0.71)	14%***	15
LB5	3.55 (0.73)	3.87 (0.66)	8%**	9
LB6	5.08 (1.07)	5.76 (0.86)	12%***	16
LB7+8	4.66 (1.36)	5.29 (1.18)	11%***	10
LB9	4.66 (1.46)	5.14 (1.32)	11%**	8
LB10	4.63 (0.99)	5.20 (1.01)	11%***	12
RB1	4.24 (0.99)	4.79 (1.11)	11%**	12
RB2	4.18 (0.87)	4.83 (0.87)	14%***	11
RB3	4.66 (1.12)	5.34 (0.85)	13%***	13
RB4	3.94 (0.99)	4.61 (1.03)	14%***	15
RB5	3.42 (1.08)	4.07 (0.73)	16%**	15
RB6	4.29 (0.92)	5.09 (0.89)	14%***	18
RB7	3.77 (0.90)	4.37 (0.90)	13%***	9
RB8	4.21 (0.88)	4.63 (0.82)	7%*	6
RB9	4.38 (0.89)	5.20 (0.80)	16%***	7
RB10	4.96 (0.86)	5.60 (0.81)	10%***	11
*Mean value*	4.21	4.81	12%	

Data are presented as mean (SD). *p<0.5; **p<0.01;***p<0.001.

**Table 3:** The changes of chest CT-estimated lobar bronchial luminal Din between acute exacerbation and stable phase of asthma.

Lobar Bronchi	Exacerbation	Stable Phase	Reduction of Din (%)	Incidence of Stenosis
LMB	11.43 (1.59)	11.96 (1.49)	5%***	12
RMB	13.99 (1.97)	14.56 (2.06)	3%***	10
LSLB	8.81 (1.52)	9.23 (1.12)	5%*	9
LILB	8.42 (1.41)	9.11 (1.16)	8%***	12
RSLB	8.04 (1.06)	8.50 (0.98)	5%***	9
IB	10.51 (1.33)	10.95 (1.33)	4%***	5
SDB	6.71 (1.32)	7.12 (0.92)	5%*	12
LB	5.50 (1.08)	6.14 (0.71)	10%***	18
RMLB	6.08 (1.34)	6.70 (1.16)	8%***	15
RILB	7.96 (1.51)	8.75 (1.35)	9%***	15
*Mean value*	8.74	9.3	6%	

Data are presented as mean (SD). *p<0.5; **p<0.01;***p<0.001.

## Data Availability

All the data supporting our findings are included in the manuscript.

## LIST OF ABBREVIATIONS

Din: Diameter of the airway
HRCT: High-resolution CT
PFTs: Pulmonary function tests
FEV1: Forced expiratory volume in one second
FVC: Forced vital capacity
WA: Wall area
BSA: Body surface area

## STANDARDS OF REPORTING

STROBE guidelines were followed.
